# Bilateral mantle cell lymphoma of the ciliary body that responded to a combined local radiotherapy and chemotherapy regimen: a case report

**DOI:** 10.1186/s12885-019-5530-7

**Published:** 2019-04-15

**Authors:** Minghang Pei, Chan Zhao, Fei Gao, Meifen Zhang

**Affiliations:** Department of Ophthalmology, Peking Union Medical College Hospital, Chinese Academy of Medical Sciences and Peking Union Medical College, Beijing, China

**Keywords:** Mantle cell lymphoma, Ciliary body, Non-Hodgkin lymphoma, Ultrasound biomicroscopy

## Abstract

**Background:**

Mantle cell lymphoma (MCL) is a rare, aggressive B-cell non-Hodgkin lymphoma (NHL) that often affects men over the age of 60. Systemic metastasis of MCL to eyes is rare and intraocular involvement is even rarer, which usually affects the choroid and iris. To the best of our knowledge, ciliary body metastasis of systemic MCL has not been reported.

**Case presentation:**

A 59-year-old Han Chinese male with past-history of systemic MCL complained of redness, pain and blurred vision in the left eye. Ocular examination revealed a normal appearance in the right eye, and conjunctival injection, pseudohypopyon and anterior protrusion of peripheral iris in the left eye, all of which were unresponsive to corticosteroid treatments. Ultrasound biomicroscopy (UBM) and B-scan were then performed which detected ciliary body masses in both eyes with no vitreous and retino-choroidal anomalies. Combined liquid-based cytology tests and gene rearrangement assays of the aqueous humor specimen confirmed this to be a B-cell malignancy. Then both eyes were treated with external beam irradiation (40 Gy, delivered evenly in twenty fractions) over a course of one month. Additionally, the left eye received intravitreal methotrexate (MTX) (weekly for the first month, every two weeks for the second month, and monthly thereafter) over a course of twelve months. This therapy eventually led to complete remission of all symptoms in one month and disappearance of the ciliary body masses in twelve months.

**Conclusion:**

Here we first reported a case of bilateral ciliary body MCL infiltration which was diagnosed by combined liguid-based cytology and gene rearrangement of aqueous humor cells. UBM may serve as a valuable tool in the diagnosis and serial assessments of anterior segment tumors.

## Background

Mantle cell lymphoma (MCL) is a rare, B-cell non-Hodgkin lymphoma (NHL) that most often affects men over the age of 60. It comprises 6 to 8% of all non-Hodgkin lymphomas with an annual incidence of 0.4 per 100,000 persons in the United States and Europe [1]. The disease is called “mantle cell lymphoma” because the tumor cells originally came from the “mantle zone” of the lymph node. As an aggressive form of NHL, MCL is resistant to conventional chemotherapeutic regimens with a tendency of multiple relapses, which results in a poor median survival of 3–6 years after diagnosis [2–4]. However, systemic metastasis of MCL to eyes is rare with most cases confined to the orbit and adnexa, intraocular involvement is even rarer, and it usually affects the choroid and iris according to the seven cases reported [5–11]. To the best of our knowledge, ciliary body metastasis of systemic MCL has not been reported.

## Case presentation

A 59-year-old Han Chinese male was diagnosed with systemic MCL in February 2014. He received three cycles of cyclophosphamide, doxorubicin, vincristine, prednisone (CHOP) and rituximab chemotherapy, one cycle of rituximab mega-CHOP chemotherapy, and two cycles of dexamethasone, cytarabine, cisplatin (DHAP) and rituximab chemotherapy, followed by BCNU, etoposide, cytarabine and melphalan (BEAM) chemotherapy and autologous stem cell transplantation (ASCT) in August 2015, which result in complete disease remission with negative restaging positron emission tomography (PET) scan obtained in November 2015.

**Fig. 1:**
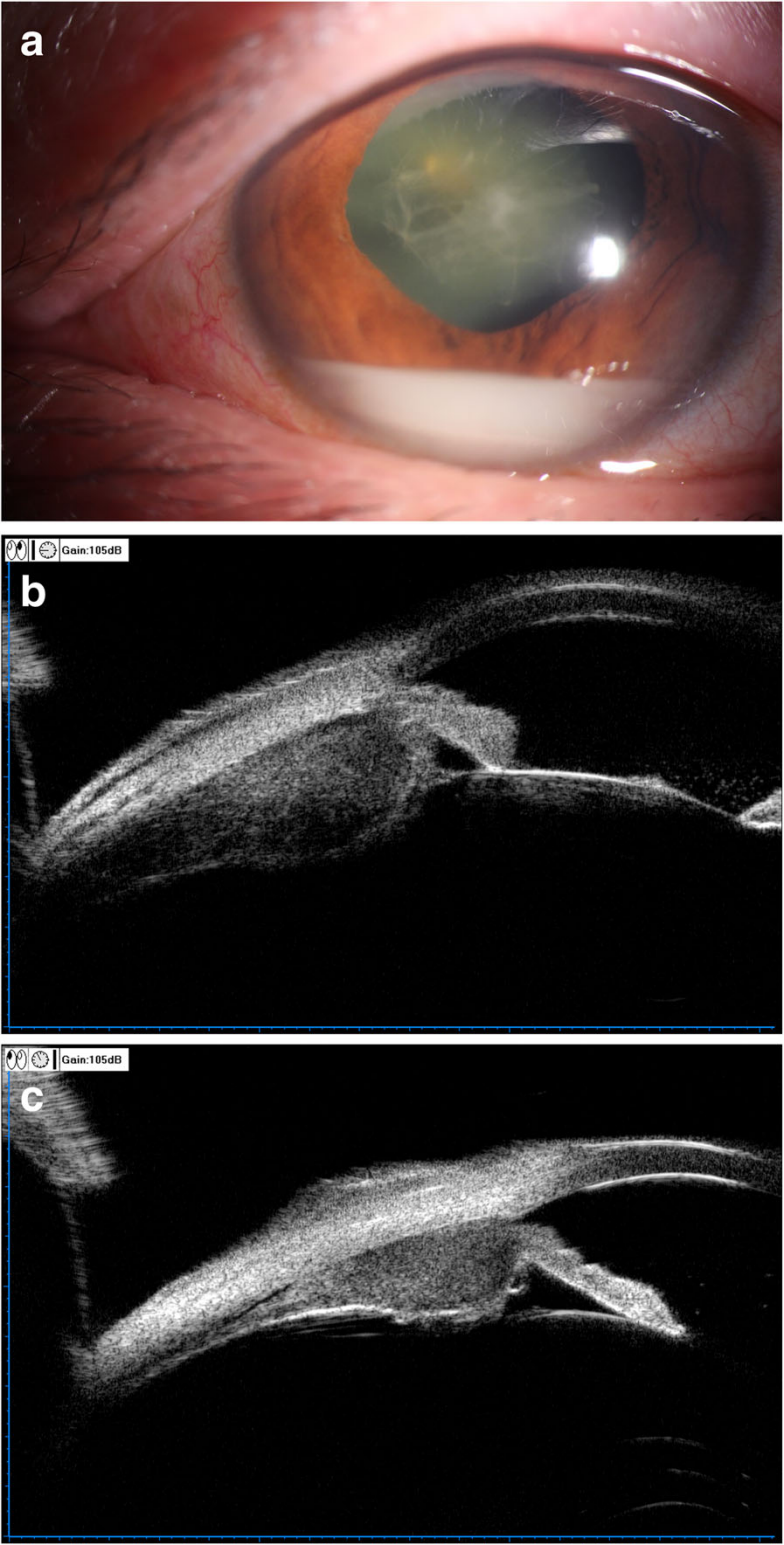
: **a** Slit-lamp photograph (**a**) showing mantle cell lymphoma (MCL) involving the anterior chamber (AC). There was diffuse conjunctival injection, pseudohypopyon and fibrin over the pupil. **b** Ultrasound biomicroscopy (UBM) showing representative ciliary masses in the left eye. **c** Ultrasound biomicroscopy (UBM) showing representative ciliary masses in the right eye

In March 2016, the patient complained of redness, pain and blurred vision in the left eye. Ocular examination revealed elevated intraocular pressure (IOP, 24 mmHg), diffuse conjunctival injection and anterior chamber reaction (flare 1+, cell 1+) in the left eye, and cortical cataracts in both eyes. The best corrected visual acuity (BCVA) was 20/50 and 20/60 in his right and left eye respectively. He was diagnosed as “anterior uveitis” and was given 1% prednisone acetate eye drops 8 times daily with tapering, along with topical tropicamide and 2% cartelol eye drops. However, the condition of his left eye continued to deteriorate which presented as “pseudohypopyon” and anterior protrusion of the peripheral iris 2 months later (Fig. 1a), and his left BCVA dropped to hand motion. Ultrasound biomicroscopy (UBM) revealed confluent ciliary body masses with almost 360°involvement in both eyes (Fig. 1b,c), while ultrasound B-scan demonstrated a clear vitreous cavity and the absence of retino-choroidal anomalies. Contrast-enhanced magnetic resonance imaging (MRI) revealed no significant findings in the orbit.

**Fig. 2:**
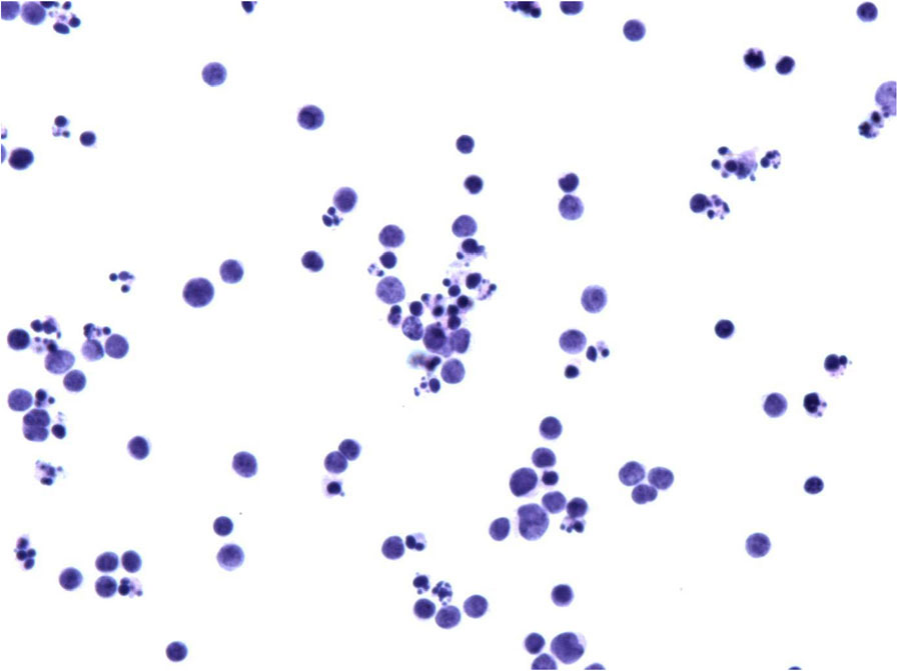
Liquid-based cytology test of the aqueous humor

Diagnostic paracentesis of the left anterior chamber was performed on April 29th 2016. Liquid-based cytology revealed small malignant cells in the aqueous humor (Fig. 2), which were then confirmed to be B-cell in origin by gene rearrangement studies. Given his past medical history, intraocular MCL was diagnosed. A PET examination was re-performed in May 2016 and no signs of systemic recurrence was identified.

**Fig. 3:**
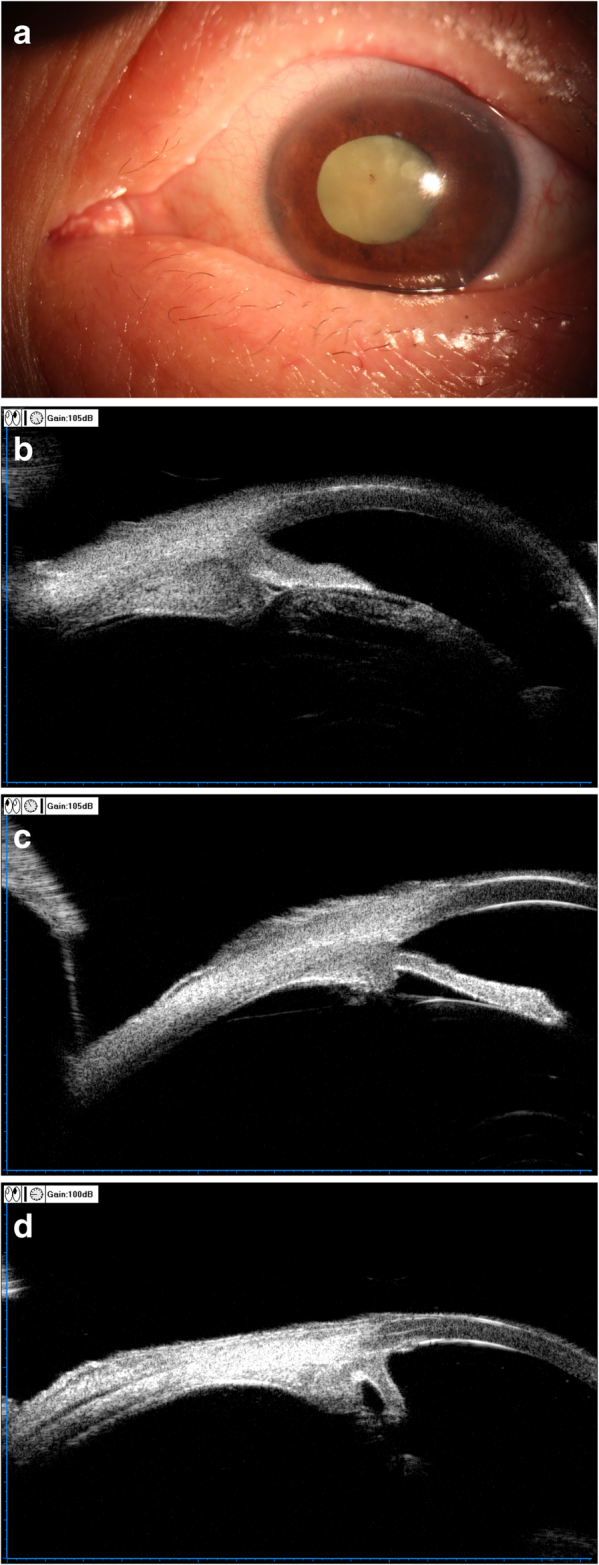
**a** Slit-lamp photograph taken after orbital radiotherapy showed a marked decrease in conjunctival injection, disappearance of pseudohypopyon and a deterioration of cataract in the left eye. **b** Ultrasound biomicroscopy (UBM) re-examination showed markedly decreased ciliary tumor sizes in the left eye. **c** Ultrasound biomicroscopy (UBM) re-examination showed complete disappearance of ciliary tumor in the right eye after orbital radiotherapy. **d** Ultrasound biomicroscopy (UBM) re-examination showed complete disappearance of ciliary tumor in the left eye after 12 months of MTX intravitreal injections

Then he was scheduled for 40 Gy external beam irradiation delivered evenly in twenty fractions over a period of 1 month. The pseudohypopyon disappeared 2 weeks after the commencement of irradiation and the symptoms resolved completely after the patient received full irradiation dosage (Fig. 3a). UBM re-examination disclosed complete regression of ciliary tumor in the right eye (Fig. 3b) and reduction of tumor size in the left eye (Fig. 3c). Additionally, his left eye received a series of 400μg/0.1 ml methotrexate (MTX) intravitreal injections, weekly for the first month, every 2 weeks for the second month, and monthly for 10 months [12], which resulted in complete regression of ciliary tumor (Fig. 3d). In January 2017, he underwent cataract surgery in the left eye with insertion of an AcrySof IQ intraocular lens, and the BCVA improved to 20/200.

Unfortunately, the patient suffered peripheral lymph node MCL recurrence in October 2018, which followed by rapid deterioration. In November 2018, he died of the side effects of intensive chemotherapy.

## Discussion and conclusions

MCL, which constitutes 6% of all non-Hodgkin lymphoma (NHL), is a mature but aggressive B-cell lymphoma with a propensity for widespread dissemination, including lymphoreticular and gastrointestinal metastasis [6]. Ocular adnexal involvement of all NHL is clinically detectable in about 1.3–5% of cases, of which 5% have been found to be secondary to MCL. Intraocular involvement of MCL, notably, is extremely rare and has only been reported in 7 cases in the literature [5].

Isolated involvement of the anterior segment was observed in 3 of the 7 intraocular MCL cases. In a report by Economou et al., bilateral involvement of the iris was noted in a 71-year-old male with large, reddish tumor-like masses, anterior chamber inflammation, and hyphema [8]. Reid et al. reported unilateral granulomatous anterior chamber inflammation and localized undulations of the iris with aberrant vasculature in an elderly male of systemic MCL [9]. Agarwal et al. reported another MCL case presented with unilateral anterior chamber hypopyon, thickening of the iris and iris neovascularization [5]. Although malignancy was believed to mainly involve the iris in these cases [5, 7–9], ciliary body was the major site of MCL recurrence in our case as revealed by UBM, despite that a concomitant anterior chamber reaction was also observed.

Evidences for diagnosis of intraocular MCL were variable in the reported cases. In most of the cases [6–8], intraocular malignancy was established solely on ocular manifestation and past history of systemic MCL, while two cases had more solid flow cytometric evidences [5, 9] and only one patient had cytopathologic confirmation [7]. In our case, intraocular involvement of MCL was confirmed by both liquid-based cytology and gene rearrangement of aqueous humor cells, which to the best of our knowledge has not been reported previously in the clinical setting of intraocular MCL.

UBM produced high-resolution images of the ciliary body resulting in excellent visualization of the tumor location and measurement of the tumor dimensions in our patient. Careful evaluation of morphological features of the ciliary body on UBM enabled us an effective monitoring of the treatment response and an objective assessment of the reduction in tumor load.

In summary, this is the first case report describing bilateral ciliary body infiltration of MCL. Liquid-based cytology and gene rearrangement of aqueous humor cells represent a viable diagnostic option for intraocular neoplasms with prominent anterior chamber reaction. And UBM may serve as a valuable tool in the diagnosis and serial assessments of anterior segment tumors.

## Abbreviations

ASCT: Autologous stem cell transplantation
BCVA: Best corrected visual acuity
BEAM: BCNU, etoposide, cytarabine and melphalan
CHOP: Cyclophosphamide, doxorubicin, vincristine, and prednisone
DHAP: Dexamethasone, Cytarabine, Cisplatin
IOP: Intraocular pressure
MCL: Mantle cell lymphoma
MRI: Magnetic resonance imaging
MTX: Methotrexate
NHL: non-Hodgkin lymphoma
PET: Positron emission tomography
UBM: Ultrasound biomicroscopy
